# Psoas Abscess Caused by Candida Glabrata: A Case Report

**DOI:** 10.7759/cureus.10614

**Published:** 2020-09-23

**Authors:** Sandhya Nagarakanti, Eliahu Bishburg

**Affiliations:** 1 Internal Medicine/Infectious Disease, Newark Beth Israel Medical Center, Newark, USA

**Keywords:** candida glabrata, psoas abscess

## Abstract

Psoas abscess caused by Candida is an uncommon condition. We report a case of psoas abscess caused by Candida glabrata, which was completely resolved with drainage and oral voriconazole. Because of the nonspecific clinical presentation, the diagnosis of psoas abscess can be a challenge. Prompt suspicion, with early diagnosis and drainage with an appropriate antifungal agent, seems to improve the clinical outcome.

## Introduction

Psoas abscess is an uncommonly encountered clinical entity [1]. It could spread from an adjacent anatomical area, such as a vertebral abscess, gastrointestinal or genitourinary focus, or occasionally seeding from a hematogenous infection [2]. Patients commonly present with nonspecific symptoms such as abdominal pain [3]. A computed tomogram of the abdomen is used to diagnose the condition. The most commonly implicated organisms are *Staphylococcus aureus *and *Mycobacterium tuberculosis (M. tuberculosis) *[4-6]. Psoas abscess caused by Candida species has only rarely been described.

## Case presentation

A 35-year-old female was admitted with fever, dysuria, and suprapubic pain for one week. Her past medical history was significant for diabetes mellitus (DM) and stage 1 B endometrial carcinoma for which she underwent a robotic-assisted laparoscopic hysterectomy, with bilateral salpingo-oophorectomy and lymph node dissection, four months prior to the admission.

Vital signs on admission revealed a temperature of 102.7F, blood pressure 111/70 mm/Hg, heart rate 107/minute, respiratory rate 18/minute, with oxygen saturation of 93% on room air. There was tenderness over the suprapubic and right-sided lumbar area with a full range of motion of the lower extremities.

Laboratory data revealed a white blood cell count of 6X103/µL, hemoglobin 9.7 g/dL (Normal range=12 to 16.3 g/dL), platelet count 365 X103/µL (Normal range=150-450X103/µL), creatinine 0.59 mg/dl (Normal range=0.7-1.3 mg/dl), erythrocyte sedimentation rate 100 mm/hour (Normal range=0-15 mm/hour), and C-reactive protein 27.10 mg/dl(Normal range< 0.29 mg/dl). Urinalysis revealed trace protein, with positive leukocyte esterase, negative nitrite and white blood cells of 240, trace protein. The patient was empirically started on intravenous vancomycin 1 gram every 12 hours and intravenous piperacillin/tazobactam 3.375 grams every eight hours for a presumed urinary tract infection. 

**Figure 1:**
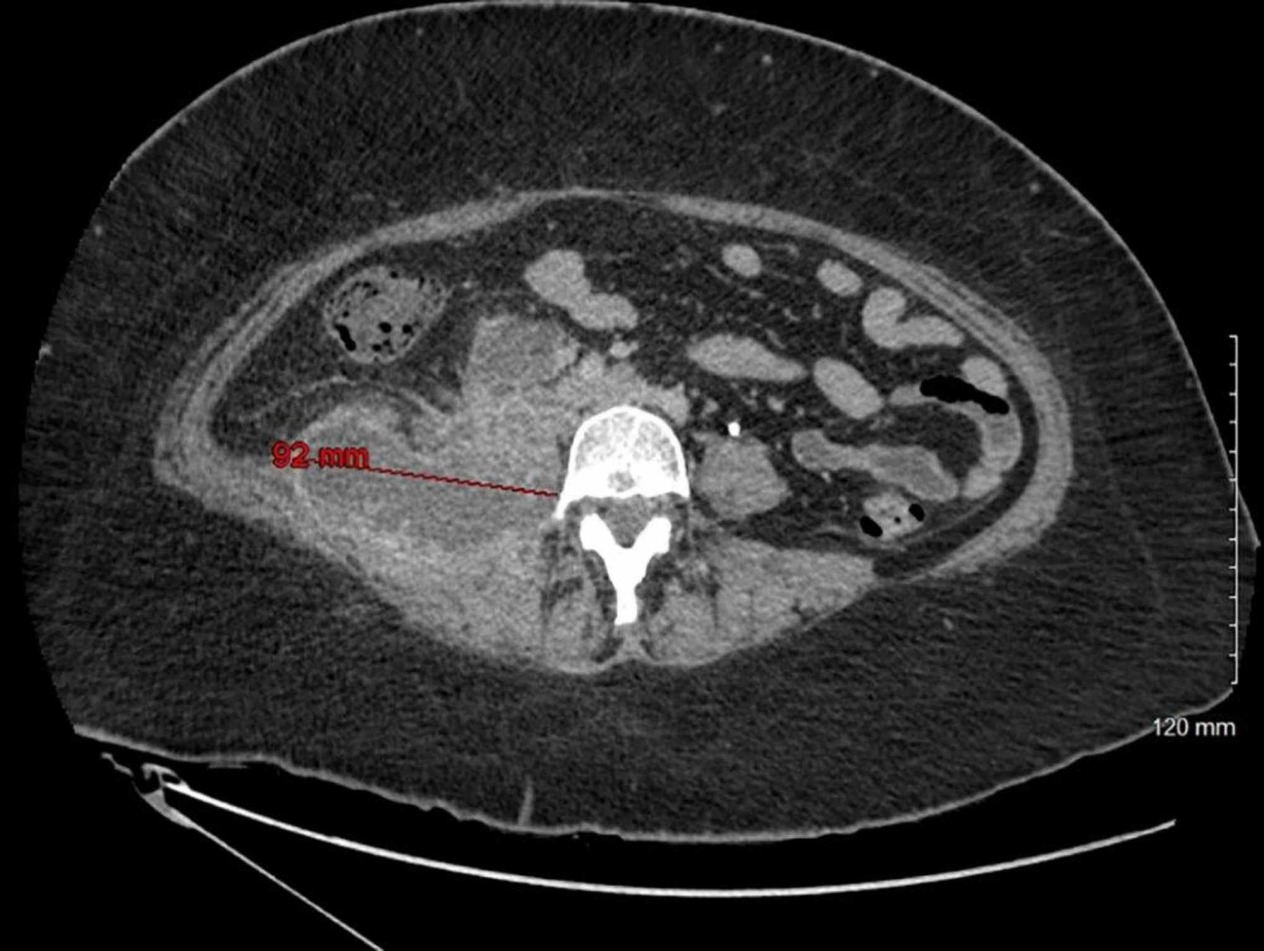
CT abdomen/pelvis with contrast on admission Multiloculated abscess 8 x 9 x 9 cm in the right psoas and quadratus lumborum musculature CT: computed tomography

**Figure 2:**
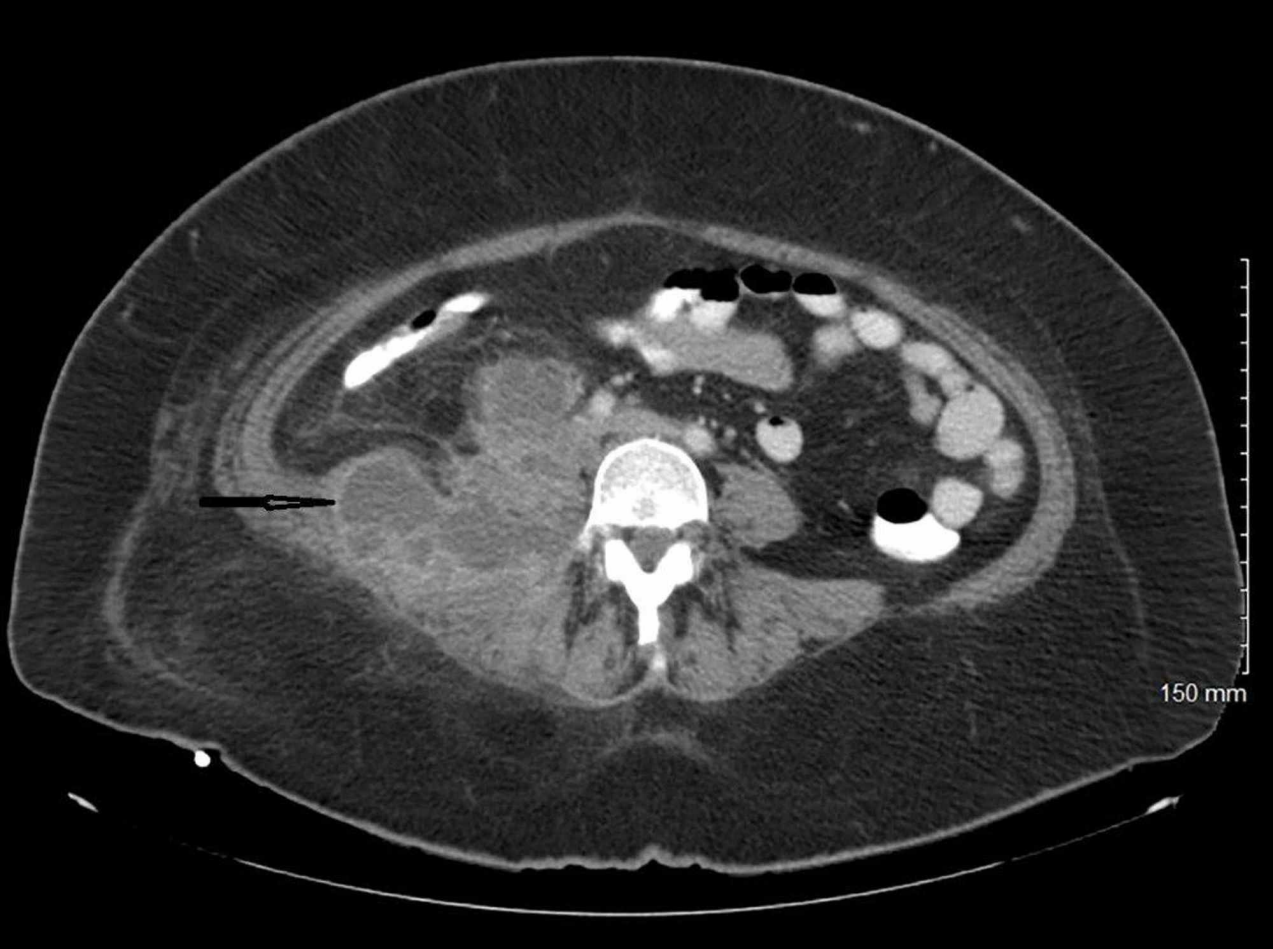
CT abdomen and pelvis with contrast after drainage and two days of antifungals Abscess in the right psoas and paravertebral muscles measuring 8 x 9 cm x 8 cm extending posterolaterally to involve the right external and internal oblique muscles CT: computed tomography

**Figure 3:**
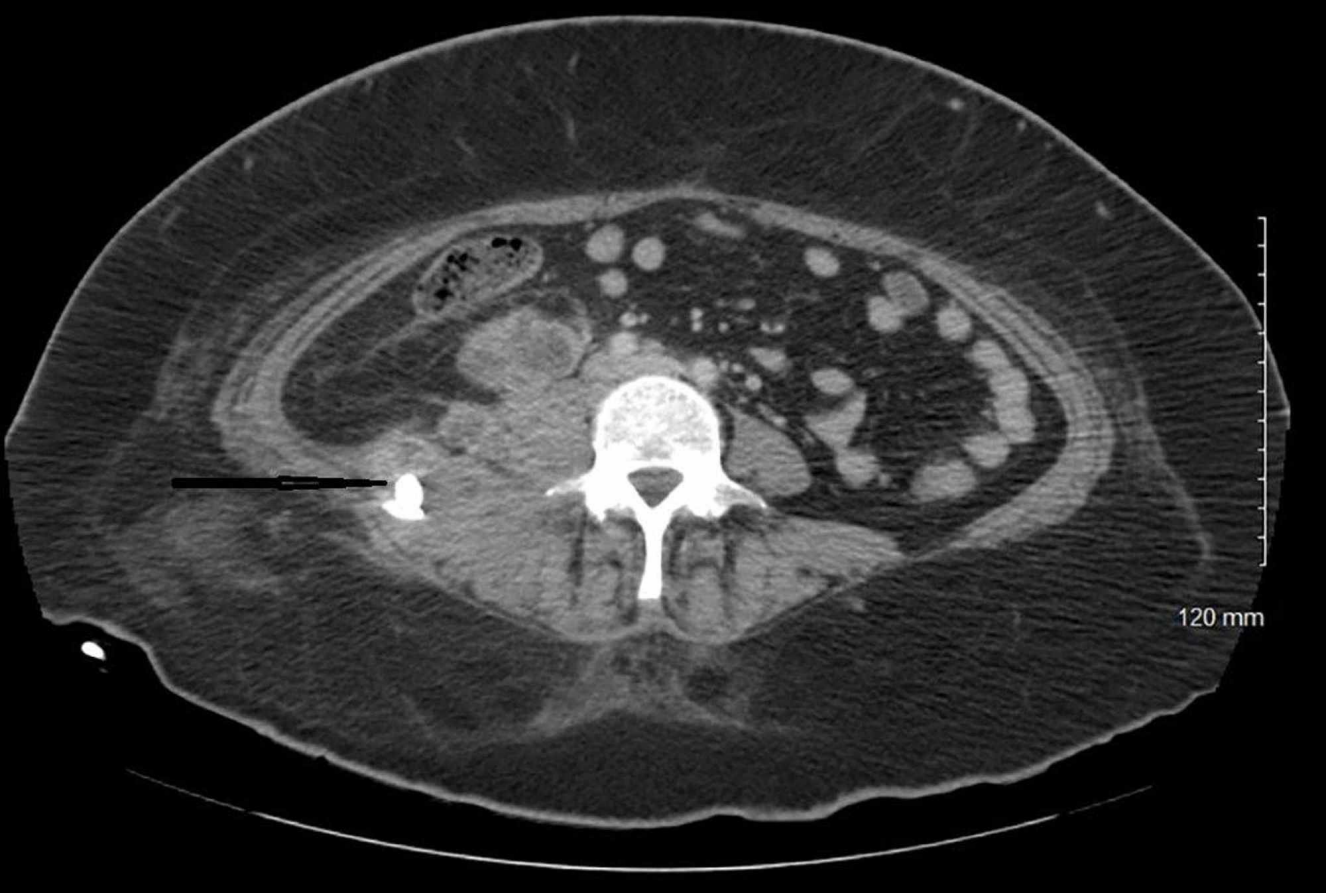
CT abdomen and pelvis with contrast after drainage and six days of antifungals Interval decrease in the size of the abscess at the site of the pigtail catheter placement CT: computed tomography

**Figure 4:**
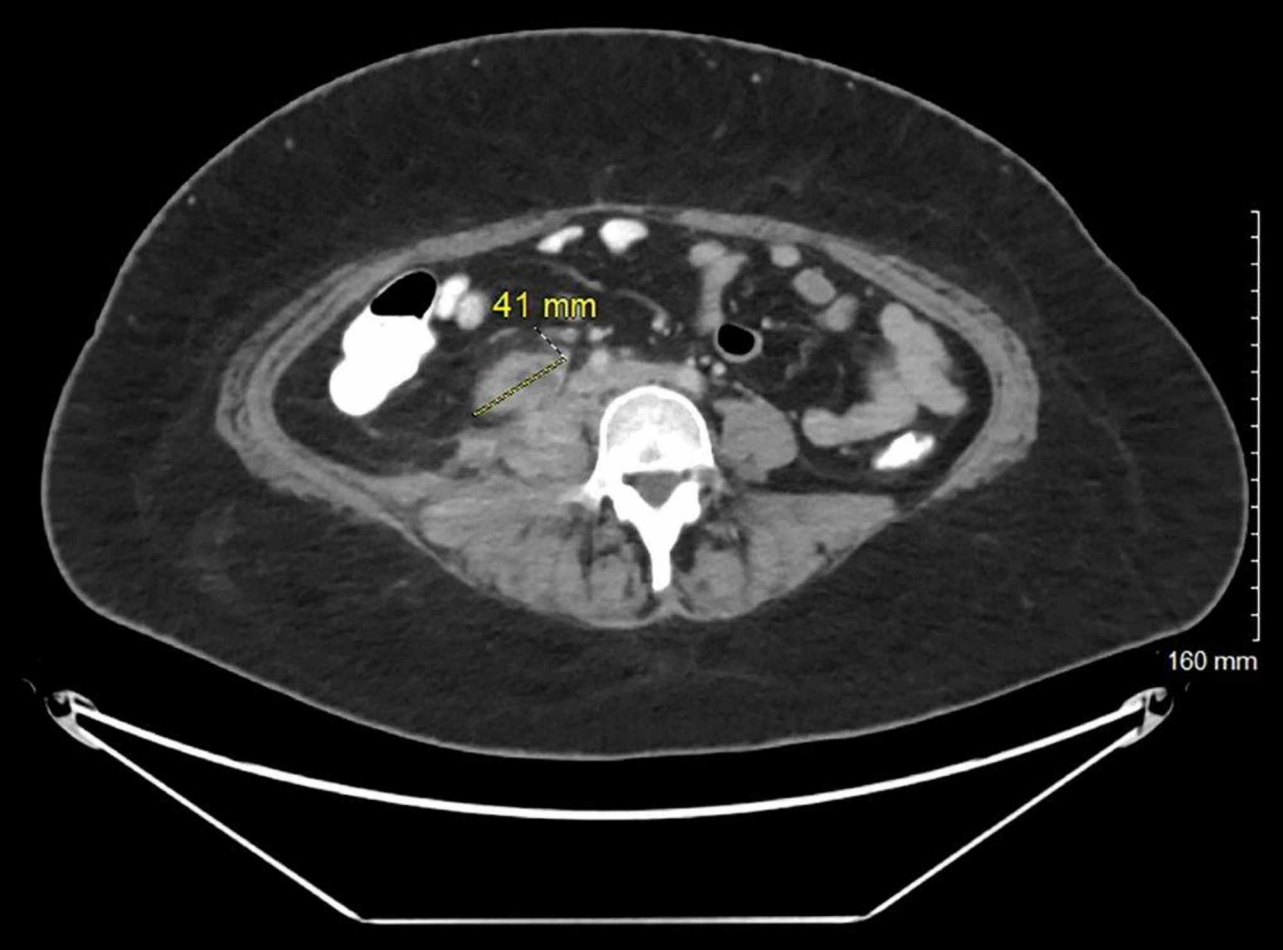
CT abdomen/pelvis with contrast at four weeks Complete resolution of the psoas abscess, a necrotic right retroperitoneal mass measuring approximately 4.1 cm, which is inseparable from the right iliopsoas muscle, which is consistent with metastatic disease CT: computed tomography

The patient underwent computed tomography (CT) of the abdomen/pelvis with contrast, which revealed multiloculated abscess 8 x 9 x 9 cm in the right psoas and quadratus lumborum musculature, however, an underlying necrotic tumor could not be excluded (Figure 1). The patient underwent ultrasound-guided drainage with the placement of a pigtail catheter and the removal of 25 cc of purulent fluid. Gram stain of the fluid showed many white cells and rare yeast. Blood cultures were negative and urine cultures grew *Escherichia coli (E. coli) *sensitive to ceftriaxone, cefazolin, ceftazidime, nitrofurantoin, and piperacillin/tazobactam. Vancomycin was discontinued, and the patient was continued on piperacillin/tazobactam to complete a seven-day course for a urinary tract infection. Culture of the drained abscess grew Candida glabrata in pure culture. The organism was sensitive to fluconazole with a minimum inhibitory concentration (MIC) of <2 µg, caspofungin MIC < 0.25 µg, and voriconazole MIC <0.12 µg. The patient was started on intravenous fluconazole 400 mg every 24 hours based on susceptibility to fluconazole. Repeat CT of the abdomen/pelvis with contrast was performed two days after starting intravenous fluconazole, which showed an increase in the size of the heterogeneous collection within the right psoas and paravertebral muscles, with its largest component measuring 8 x 9 x 8 cm. This collection extended posterolaterally to involve the right external and internal oblique muscles and more of the right paravertebral musculature. There was overall increased fat stranding within this region. This mass also extended anteriorly with a component just inferior to the lower pole of the right kidney, measuring approximately 3 x 4 x 4 cm; the drainage catheter, with its tip, was seen within posterior right subcutaneous fat (Figure 2). The patient underwent new ultrasound-guided drainage of the abscess with the removal of 30 cc of purulent fluid and placement of a new pigtail catheter with the removal of the old drain. Intravenous fluconazole was changed to intravenous caspofungin, as the CT abdomen showed an increase in the collection, as susceptibility in-vitro does not always predict the clinical response. The patient continued on intravenous caspofungin for six days. Repeat CT of the abdomen showed an interval decrease in the size of the abscess at the site of the pigtail catheter placement and an interval increase in the size of 4.5 x 2.3 cm abscess in the subcutaneous fat in the right flank superficial to the drainage catheter (Figure 3). The pigtail catheter was removed and intravenous caspofungin was changed to oral voriconazole 200 mg twice daily, and the patient was discharged home. The serum voriconazole level was maintained at 3.1 µg/ml. The patient remained asymptomatic and underwent another CT abdomen four weeks later, which showed complete resolution of the psoas abscess, a necrotic right retroperitoneal mass measuring approximately 4.1 cm, which is inseparable from the right iliopsoas muscle, which is consistent with metastatic disease. There were additional smaller adjacent lymph nodes and no free intraperitoneal air or loculated fluid collections (Figure 4).

## Discussion

Psoas abscess is a rare condition with a reported worldwide incidence of 12 new cases per year [1]. The condition was first described by Mynter in 1881 as psoitis: “a collection of pus in the Psoas muscle” [2]. The psoas muscle courses from the lower part of the thorax to the lower lumbar vertebrae and femur through the retroperitoneum [3]. The muscle has a rich blood supply, making it susceptible to the hematogenous seeding of bacteria, and its anatomical closeness to the gastrointestinal system and spinal column, making it susceptible to infections via direct spread from these sites [4-5].

The most commonly described conditions associated with psoas abscess include vertebral osteomyelitis, epidural abscess, septic arthritis, discitis, sacroiliitis, gastrointestinal diseases, such as Crohn's disease, appendicitis, pancreatitis, infected aortic aneurysms, rectal surgery, and nephrectomy, and immunosuppressive conditions such as malignancy, organ transplantation, human immunodeficiency syndrome (HIV) infection, and tuberculosis of the spine (Pott’s disease) [5-7].

Most patients with psoas abscess present with nonspecific symptoms, such as abdominal pain. Patients commonly have an elevated white blood cell (WBC) count and elevated inflammatory markers such as erythrocyte sedimentation rate and C-reactive protein [6]. CT of the abdomen is the most common modality to diagnose psoas abscess. Tabrizian et al. reported that the mean size of the abscess was 6 cm at diagnosis, the abscesses were unilateral in over 50% of the patients and multiple in 25% of the patients [6]. The most common etiology of psoas abscess is *Staphylococcus aureus*, followed by *Streptococci* and *E. coli* [5,8-9]. *M. tuberculosis* was the most common isolate in another psoas abscess case series [7].

Candida sp. is a rare pathogen causing psoas abscesses.* Candida albicans* is reported as a cause of psoas abscess; one case reported a 59-year-old female with diabetes mellitus who presented with a right flank mass of which an aspirate grew *Candida albicans* [10]. *Candida glabrata* as a cause of psoas abscess was reported in two cases, both in the non-English language literature [11-14]. The first case was reported in one out of 38 psoas abscess cases reviewed by Hammami et al. [15]. A second case was described in a 51-year-old diabetic male who was admitted with hip pain and fever, the patient was diagnosed with a psoas abscess with the isolation of *Candida glabrata *from the abscess. The patient improved after percutaneous drainage and oral voriconazole [16].

Our patient's case is similar to the case described by Kim et al. [16], in that it occurred in a patient with underlying DM when both cases improved after percutaneous drainage and voriconazole treatment.

The successful treatment of psoas abscess consists of drainage of the abscess along with systemic antifungal agents [6]. In some cases, surgical drainage is required, and this approach has been shown to have a higher success rate in the treatment than percutaneous drainage [8].

It is not clear why our patient developed this rare condition but as mentioned previously, she had previously described risk factors such as DM as well as endometrial cancer.

## Conclusions

To our knowledge, our case is the first case of* Candida glabrata* psoas abscess described in the English literature. Because of the nonspecific clinical presentation, the diagnosis of psoas abscess can be a challenge to the clinician. Early diagnosis with prompt drainage with appropriate antifungal agent seems to improve outcomes.
